# Neurological and psychiatric disorders among autistic adults: a population healthcare record study

**DOI:** 10.1017/S0033291722002884

**Published:** 2023-09

**Authors:** Jack F. G. Underwood, Marcos DelPozo-Banos, Aura Frizzati, Dheeraj Rai, Ann John, Jeremy Hall

**Affiliations:** 1 Division of Psychological Medicine and Clinical Neurosciences, Neuroscience and Mental Health Innovation Institute, Cardiff University, Cardiff, UK; 2 Population Data Science, Medical School, Swansea University, Swansea, UK; 3 Cedar Healthcare Technology Research Centre, Cardiff & Vale University Health Board, Cardiff, UK; 4 Bristol Medical School, Bristol Population Health Science Institute, Bristol, UK

**Keywords:** Autism spectrum disorders, autism, co-morbidity, co-occurring conditions, epidemiology

## Abstract

**Background:**

Co-occurring psychiatric disorders are common in autism, with previous studies suggesting 54–94% of autistic individuals develop a mental health condition in their lifetime. Most studies have looked at clinically-recruited cohorts, or paediatric cohorts followed into adulthood, with less known about the autistic community at a population level. We therefore studied the prevalence of co-occurring psychiatric and neurological conditions in autistic individuals in a national sample.

**Methods:**

This retrospective case-control study utilised the SAIL Databank to examine anonymised whole population electronic health record data from 2001 to 2016 in Wales, UK (*N* = 3.6 million). We investigated the prevalence of co-occurring psychiatric and selected neurological diagnoses in autistic adults' records during the study period using International Classification of Diseases-10 and Read v2 clinical codes compared to general population controls matched for age, sex and deprivation

**Results:**

All psychiatric conditions examined were more common amongst adults with autism after adjusting for age, sex and deprivation. Prevalence of attention-deficit hyperactivity disorder (7.00%), bipolar disorder (2.50%), obsessive-compulsive disorder (3.02%), psychosis (18.30%) and schizophrenia (5.20%) were markedly elevated in those with autism, with corresponding odds ratios 8.24–10.74 times the general population. Depression (25.90%) and anxiety (22.40%) were also more prevalent, with epilepsy 9.21 times more common in autism.

**Conclusions:**

We found that a range of psychiatric conditions were more frequently recorded in autistic individuals. We add to understanding of under-reporting and diagnostic overshadowing in autism. With increasing awareness of autism, services should be cognisant of the psychiatric conditions that frequently co-occur in this population.

## Introduction

Autism spectrum disorder [henceforth ‘autism’, in combination with identity-first language reflecting community preference (Bury, Jellett, Spoor, & Hedley, 2020; Kenny et al., 2015)] is a complex neurodevelopmental condition characterised by persistent difficulties in social interaction and communication, stereotypic, repetitive and restricted behaviours and interests (World Health Organisation, 2018). Autism is common, with epidemiological studies reporting a prevalence of 1% in the population, and a male to female ratio of around 3:1 (Brugha et al., 2011, 2016; Chiarotti & Venerosi, 2020; Fombonne, MacFarlane, & Salem, 2021). Studies of predominantly clinical environment recruited samples suggest between 54% and 94% of autistic adults will experience another co-occurring mental health disorder in their lifetime, with associated morbidity and detriment to quality of life (Hossain et al., 2020; Pehlivanidis et al., 2020). Amongst the general population the prevalence of co-occurring mental health disorders in autistic adults is not clear and diagnostic overshadowing within healthcare is of particular concern (Kerns et al., 2015; Lai et al., 2019).

The majority of studies of autistic individuals have been conducted in paediatric populations. These have demonstrated higher prevalences of a number of co-occurring psychiatric and physical health disorders, including anxiety, depression, attention-deficit hyperactivity disorder (ADHD), oppositional defiant disorder, seizures, sleep disorders, gastrointestinal disorders and metabolic disorders (Bauman, 2010; Bradley & Bolton, 2006; Hossain et al., 2020; Levy et al., 2010; Lugnegård, Hallerbäck, & Gillberg, 2011; Pehlivanidis et al., 2020; Schieve et al., 2012; Simonoff et al., 2008; Wang et al., 2009). Studies in populations of autistic adults show this elevated risk continues into later life, with a 2018 meta-analysis demonstrating lifetime prevalence estimates of 42% for anxiety and 37% for depression (Buck et al., 2014; Hollocks, Lerh, Magiati, Meiser-Stedman, & Brugha, 2019; Joshi et al., 2013; Lugnegård et al., 2011). This analysis found considerable heterogeneity in study designs, with population-based autistic cohorts generally lacking in the literature (Hollocks et al., 2019). A further 2019 meta-analysis and systematic review by Lai et al. identified a small number of population and registry-based studies and produced pooled risk estimates (Croen et al., 2015; Kirsch et al., 2020; Lai et al., 2019; Nimmo-Smith et al., 2020). Lai et al. concluded in their review that producing reliable rates was problematic due to heterogeneous study populations, designs and measures, a sentiment echoed by Hossain et al.'s umbrella review of systematic reviews which recommended population-based observational research as an area for future research (Hossain et al., 2020; Lai et al., 2019).

Studies examining what are considered comorbid physical health conditions, such as epilepsy, amongst autistic adults are rarer still. A 2021 umbrella systematic review by Rydzewska et al. identified 24 systematic reviews and meta-analysis examining physical health amongst autistic people, of which only nine examined adults (Rydzewska, Dunn, & Cooper, 2021). The long-reported association between autism and epilepsy was examined by three reviews, with epilepsy co-occurring at prevalences of between 4.7% and 34.5%, dependent on sex and intellectual disability status (Lai, Lombardo, & Baron-Cohen, 2014; Rydzewska et al., 2021). Few reviews looked at co-occurrence of other neurological disorders such as migraine headache, which have potential pathophysiological overlap and where small clinical studies have previously shown associations (Underwood et al., 2019; Vetri, 2020). The aim of this study was therefore to report population-level estimates of prevalence in adults of psychiatric and selected neurological conditions in autistic individuals, and odds ratios relative to the general population using anonymised healthcare data. Establishing the prevalence of co-occurring psychiatric and neurological disorder may identify targets for community intervention, and highlight areas of diagnostic overshadowing evident from healthcare records leading to improvements in quality of life for autistic adults.

## Methodology

### Data sources, population and settings

This was a retrospective population-based electronic case-control study using data sourced from the Welsh Secure Anonymised Information Linkage (SAIL) Databank (www.saildatabank.com) (‘SAIL Databank – The Secure Anonymised Information Linkage Databank, n.d.’). The SAIL Databank is a data repository of anonymised person-based linkable data derived from healthcare and public records. Healthcare in Wales is delivered through a primary–secondary care model, whereby primary care general practices (family practitioners) assess individuals at first instance on healthcare complaints and provide public health interventions. Where specialist investigations or interventions are required, individuals are referred onwards to specialist secondary hospital services. All services operate under the National Health Service (NHS), split into defined geographic catchment areas known as Local Health Boards. A central ‘spine’ provides personal identifier information to clinical and social care services, with general practices and hospital services having access to an integrated national electronic record system, the Welsh Clinical Portal. Clinical encounters in general practice are recorded in individual's general practice notes, which integrate with the Welsh Clinical Portal, where hospital letters and coding for hospital admissions and investigations are recorded. Participating services submit electronic primary care, hospital administration and patient health record, and central health and social care administration records to SAIL, where identifiable information is removed and records are anonymised for inclusion. The SAIL Databank therefore provides a population-scale source of individual-level anonymised healthcare record data for the epidemiological study of diagnoses. Access to data for this project was approved by the SAIL independent Information Governance Review Panel in 2018 (application 0843). The policies, processes, permissions, controls and structures used to manage the SAIL Databank have been previously described elsewhere (Ford et al., 2009; John et al., 2018; Lyons et al., 2009).

A total of 3 632 078 individuals healthcare records over the period 2000–2016 from the Welsh population were available for inclusion in the study. This included information pertaining to individuals who had been born, died or moved away from the recruitment area, representing population turnover. Clinical diagnostic records were obtained from Welsh Longitudinal General Practice dataset (WLGP) and Patient Episode Database for Wales (PEDW). The Welsh Demographic Service Dataset (WDSD) provided anonymised demographic data. This included geographic index (LSOA = Lower Layer Super Output Area index 2011, location data where areas have approximately 1500 individuals) and deprivation markers (Welsh Index of Multiple Deprivation quintile 2011). The WDSD includes anonymised demographics and general practice registration as part of an administrative register of all individuals in Wales using NHS or social care services. At the time of the analysis 79% of the Welsh population were covered in the WLGP dataset, comprising 333 (of 432, 77%) general practices in Wales. All public NHS hospitals in Wales contributed their hospital admission (inpatient and day case) data to the PEDW dataset. Autistic individuals were identified in the WLGP and PEDW datasets using Read Codes v2 and ICD (International Classification of Diseases) version 10 codes respectively. This methodology has previously been used in other psychiatric disorders and appropriately validated (Economou et al., 2012; Lloyd et al., 2015).

**Fig. 1.**
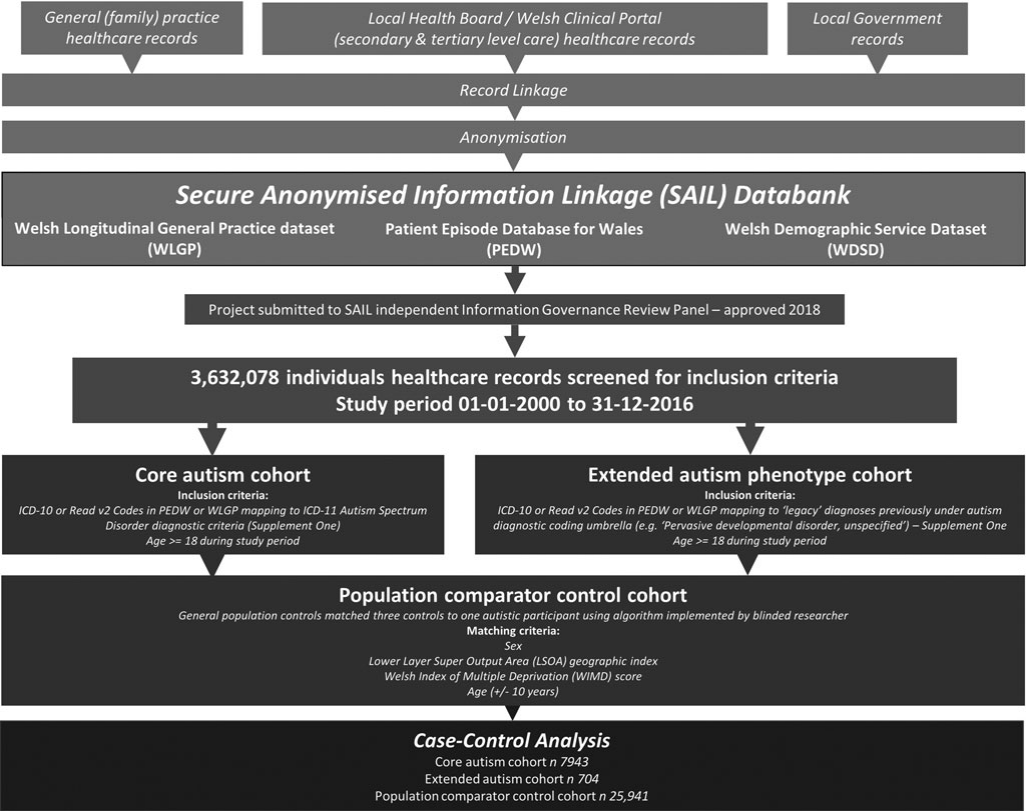
Flow chart of study methodology.

Two definitions of autism were used. The ‘core’ autism cohort utilised ICD-10 codes: F84.0, F84.1, F84.5, and Read v2 Codes: 1J9, E140, E1400, E1401, E140z, Eu840, Eu841 and Eu845. These codes directly correspond to the ICD-11 diagnoses of autism spectrum disorder, and the now deprecated ICD-10 codes including autistic disorder and Asperger's syndrome (World Health Organisation, 2018) (Fig. 1). Further individuals were identified using an ‘extended’ autism definition, which included ‘legacy’ codes utilised as part of previous work and selected for maximum sensitivity for autistic phenotypes (online Supplementary One) (Underwood, DelPozo-Banos, Frizzati, John, & Hall, 2021). These codes correspond with other less specified conditions previously considered under the ‘autistic disorder’ umbrella, such as: ‘overactive disorder associated with mental retardation and stereotyped movements’; ‘pervasive developmental disorder, unspecified’; and ‘other childhood disintegrative disorder’. This methodology has previously been used in comparable record linkage studies of autism, where the broader code definition is used to incorporate all potential presentations of autistic features (Brooks et al., 2021; Idring et al., 2015; Jariwala-Parikh et al., 2019; Skonieczna-Żydecka, Gorzkowska, Pierzak-Sominka, & Adler, 2017). Whilst these conditions are not incorporated into the latest 6A02 autism spectrum disorder coding in ICD-11, it was considered that individuals with this coding may have received such diagnostic coding due to contemporaneous presentations consistent with autism (World Health Organisation, 2018). Autism diagnostic codes were extracted across the whole lifetime, ensuring identification of all possible autistic participants.

A population comparator control cohort (the ‘population comparators’, ‘population controls’ or ‘controls’) was created by matching subjects without a record of autism in both PEDW and WLGP to autistic participants; control matches were required to have the same sex, the same LSOA geographic location and WIMD deprivation index, and a similar age (±10 years, required to accommodate the geographic and deprivation matching) at the start of the study time period (2000-01-01 to 2016-12-31) to their respective autistic participant. Where geographic/deprivation data were not available for the index participant, matched controls were selected that also lacked these data. A total of three controls were anonymously assigned to each autistic participant by algorithm, with the researchers blinded to the matching index.

The autistic and control participants were interrogated for the presence of ICD-10/Read v2 Codes referring to specific mental and neurological conditions in adult age (⩾18 years old) during the study period, examining predominantly adult diagnostic categories within the ICD-10. Follow-up period was defined as either entry into the database (for those aged over 18 at the start of the study time period) or 18th birthday. The psychiatric conditions examined were ADHD, alcohol misuse, anxiety, bipolar disorder, depression, drug misuse, obsessive-compulsive disorder (OCD), psychosis and schizophrenia based on previously validated code lists (John et al., 2020, 2016; Lee et al., 2020). Psychiatric conditions specific to childhood or adolescence were not examined. The neurological conditions examined were cluster headache, epilepsy, headache (unspecified), migraine, tension headache and vascular headache. For full ICD-10 and Read v2 Codes please see online Supplementary One.

### Statistical analysis

Data in the SAIL Databank were extracted and interrogated using code written in structured query language (SQL DB2). Analysis of outputs were processed in R version 4.0.3 (R Core Team, 2020) utilising the ‘Tidyverse’, ‘arm’, ‘effects’, ‘ResourceSelection’ and ‘gmodels’ packages (Bolker, Warnes, Lumley, & Johnson, 2018; Fox & Weisberg, 2018; Gelman & Su, 2020; Lele, Keim, & Solymos, 2019; Wickham et al., 2019). The prevalence in adults of specific psychiatric and neurological conditions in both GP and hospital records were compared, then combined, plotted and reviewed for summary statistics, with associations examined with χ^2^ tests. The vascular headache variable was excluded from further analyses due to sample size reporting restrictions within SAIL, whilst the headache (unspecified) variable was excluded due to concerns relating to diagnostic reliability, collinearity and overlap with other diagnoses. Therefore, 13 psychiatric and neurological conditions were taken forward to secondary analysis, where an adjusted binomial logistic regression model was run for each variable comparing odds between autistic participants and controls, co-varying for sex, age and deprivation (WIMD by quintile). A separate model was run for both the ‘core’ and ‘extended’ groups for each variable. Test statistics were adjusted for 30 tests using a Bonferroni correction.

## Results

### Co-occurring psychiatric conditions

There were 7943 individuals within SAIL ⩾18 years old during the 2000-01-01 to 2016-12-31 study period meeting criteria for the core autism phenotype code cohort, with an additional 704 meeting the extended cohort criteria. Autism diagnosis was recorded in WLGP records for 4906 participants of the 8647 total (56.74%), PEDW records for 1867 participants (21.59%), and was recorded in both datasets for 1874 participants (21.67%). Where autism diagnosis was recorded in both datasets, it was first recorded in the WLGP dataset in 1448 participants, in PEDW dataset for 406 participants, and in both datasets simultaneously for 20 participants. Mean age of first record of autism diagnosis was 23 years (range 10–91). This cohort was matched to 25 941 population comparator control individuals. Sex distribution was equal across the cohorts (Table 1). Mean hospital record percentage coverage was 76.86% for autistic participants, and 86.09% for control participants, with corresponding GP record percentage coverage at 65.01% and 78.77% respectively. Autism increased in prevalence in line with deprivation quintile, from 17.48% (core cohort) in the least deprived quintile to 21.80% (core cohort) in the most deprived quintile of areas.

**Table 1.** Sex, WIMD quintile and age demographics for control (general population), core and extended cohorts

		Number of core autism cases	Percentage of core autism cohort	Number of extended autism cases	Percentage of extended autism cohort	Number of population comparators	Percentage of population comparators
Gender	Male	5996	75.49	456	64.77	19 356	74.62
	Female	1947	24.51	248	35.23	6585	25.38
WIMD	1	1335	17.48	122	17.33	4371	17.50
	2	1400	18.33	126	17.90	4578	18.33
	3	1552	20.32	138	19.60	5070	20.30
	4	1687	22.08	154	21.88	5523	22.11
	5	1665	21.80	146	20.74	5433	21.75
Age	18–25	6255	78.78	514	73.01	20 970	80.87
	26–35	755	9.51	86	12.22	2270	8.75
	36–50	664	8.36	80	11.36	1995	7.69
	51–65	221	2.78	20	2.84	587	2.26
	66+	45	0.57	<5	0.57	107	0.41

Demographic details obtained from WLGP and hospital encounter data across all cohorts for individuals aged 18 or over, where matched on sex, age (±10 years) and geographic/deprivation index. WIMD, Welsh Index of Multiple Deprivation, as quintiles where 1 is least deprived and 5 is most deprived.

**Fig. 2.**
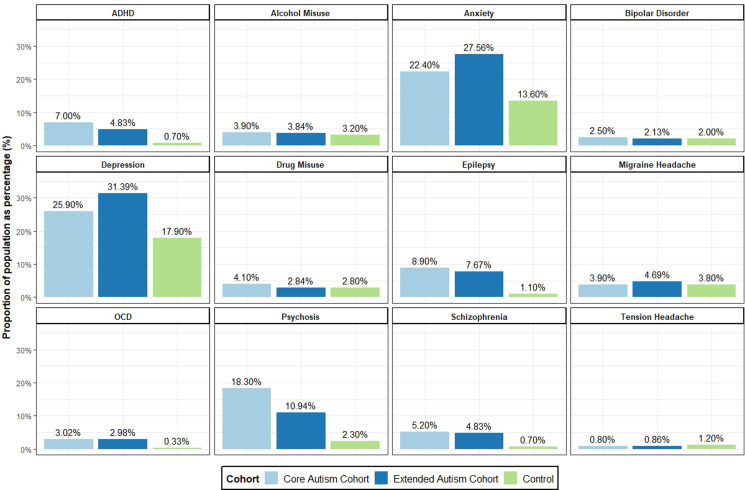
Rates of co-occurring psychiatric conditions across core autism definition, extended autism definition and population control cohorts. Crude lifetime prevalence percentages from WLGP and hospital encounter data for individuals aged 18 or over. ADHD, attention-deficit hyperactivity disorder; anxiety, generalised anxiety disorder plus phobias; OCD, obsessive-compulsive disorder. Cluster headache, vascular headache and headache (undifferentiated) not plotted due to either small sample size or non-specific sample. For specific ICD/Read v2 coding please see online Supplementary One.

Crude adult prevalence of co-occurring psychiatric conditions was elevated for all disorders compared to the control population in both the core and extended cohorts (Fig. 2, Table 2). Within the general population control sample 8707 (33.56%) individuals' records contained at least one of the studied co-occurring conditions (mental health and neurological), compared to 4364 (54.94%, χ^2^ = 1171.858, *p* < 0.001) autistic individuals in the core cohort (Table 2). Focussing on psychiatric disorders, 6607 (25.47%) population comparator individuals' records contained at least one of the nine mental health conditions examined, compared to 3783 (47.63%, χ^2^ = 1403.178, *p* < 0.001) of the core autistic cohort. Two or more of the mental health conditions were present on 2956 (11.40%) of the population comparator cohort's records, against 2067 (26.02%, χ^2^ = 1029.349, *p* < 0.001) of the core autistic individual's records. The extended autistic cohort demonstrated lower ADHD and psychosis prevalence alongside higher anxiety and depression prevalence compared to the core autism cohort on χ^2^ test, but with the same direction of effect compared to the general population comparator cohort (online Supplementary Table S1).

**Table 2.** Observed frequency count of examined mental health and neurological conditions in GP (WLGP) and hospital (PEDW) records for the core autism and general population control cohorts

	Core autism cohort				General population control cohort			
	Observed count of individuals on combined GP and hospital record		Percentage of cohort		Observed count of individuals on combined GP and hospital record		Percentage of cohort	
Any studied co-occurring condition	4364		54.94%*		8707		33.56%*	
Any studied co-occurring mental health condition	3783		47.63%*		6607		25.74%*	
Two or more co-occurring mental health conditions	2067		26.02%*		2956		11.40%*	
	Observed count of individuals with condition on GP record	Percentage of cohort with condition on GP record	Observed count of individuals with condition on hospital record	Percentage of cohort with condition on hospital record	Observed count of individuals with condition on GP record	Percentage of cohort with condition on GP record	Observed count of individuals with condition on hospital record	Percentage of cohort with condition on hospital record
Any studied mental health condition	3453	43.47%	1213	15.27%	6304	24.30%	1231	4.75%
ADHD	454	5.72%	184	2.32%	159	0.61%	47	0.18%
Alcohol misuse	227	2.86%	176	2.22%	581	2.24%	420	1.62%
Anxiety	1604	20.19%	367	4.62%	3403	13.12%	321	1.24%
Bipolar disorder	116	1.46%	135	1.70%	34	0.13%	44	0.17%
Depression	1870	23.54%	526	6.62%	4514	17.40%	550	2.12%
Drug misuse	265	3.34%	127	1.60%	591	2.28%	241	0.93%
OCD	200	2.52%	73	0.92%	81	0.31%	10	0.04%
Psychosis	1289	16.23%	434	5.46%	551	2.12%	161	0.62%
Schizophrenia	302	3.80%	256	3.22%	142	0.55%	93	0.36%
Any studied neurological condition	1284	16.17%	713	8.98%	3752	14.46%	641	2.47%
Epilepsy	384	4.83%	584	7.35%	170	0.66%	218	0.84%
Cluster headache	16	0.20%	<5	<0.06%	35	0.13%	<5	<0.02%
Migraine headache	297	3.74%	31	0.39%	954	3.68%	79	0.30%
Tension headache	62	0.78%	<5	<0.06%	298	1.15%	11	0.04%

OCD, obsessive-compulsive disorder; anxiety, generalised anxiety disorder plus phobias; ADHD, attention-deficit hyperactivity disorder; GP record, WLGP dataset; hospital record, PEDW dataset.

Raw observed count frequency and calculated percentage prevalence of examined conditions. Individuals may have diagnoses recorded on one or both sets of records.

Core autism cohort denominator 7943. Control cohort denominator 25 941. *Indicating significantly different result on χ^2^ test.

All psychiatric disorders were recorded at higher counts in the general practice (WLGP) records than in hospital admission data (PEDW) except for bipolar disorder (Table 2). For some diagnoses there were discordant patterns of recording, with anxiety and depression recorded far more frequently in general practice (WLGP) records (20.19% and 23.54% respectively) than in hospital encounter records (PEDW, 4.62% and 6.62% respectively). This pattern was not seen in diagnoses such as schizophrenia, alcohol misuse and bipolar disorder. All psychiatric disorders assessed occurred in healthcare records at elevated odds ratios in the core autism cohort when compared to the general population controls after correction for multiple testing (Table 3). Diagnoses of ADHD (core cohort OR 10.74, 95% CI 9.07–12.79, *p* < 0.001), bipolar disorder (core cohort OR 10.51, 95% CI 7.94–14.10, *p* < 0.001), OCD (core cohort OR 9.49, 95% CI 9.07–12.79, *p* < 0.001), psychosis (core cohort OR 10.15, 95% CI 9.17–11.26, *p* < 0.001) and schizophrenia (core cohort OR 8.24, 95% CI 6.89–9.90, *p* < 0.001) were strikingly increased in the health records of both autism cohorts. Alcohol misuse, anxiety, depression and drug misuse diagnoses demonstrated a still elevated but lower risk increase in the core autism cohort (Table 3). This pattern continued in the extended phenotype definition cohort, although not all associations remained significant after multiple testing in this smaller sample. Age was a significant co-variate in all mental health models, showing a linear relationship of increasing age associating with increasing risk of recorded diagnosis (online Supplementary Table S2). Deprivation was also significant except in bipolar disorder, OCD and schizophrenia, showing increasing odds of all other co-occurring mental health disorders with worsening deprivation.

**Table 3.** Associations to autism for assessed psychiatric disorders from binomial logistic regression models for each co-occurring condition

	Core autism cohort			Extended autism cohort		
	Adjusted odds ratio	95% CI	*p*	Adjusted odds ratio	95% CI	*p*
*Psychiatric conditions*						
Any studied psychiatric condition	2.83	2.68–2.99	<0.001*	2.38	2.03–2.78	<0.001*
ADHD	10.74	9.07–12.79	<0.001*	7.82	5.24–11.35	<0.001*
Alcohol misuse	1.25	1.09–1.43	0.036*	1.25	0.82–1.81	1.000
Anxiety	1.90	1.78–2.03	<0.001*	2.26	1.89–2.69	<0.001*
Bipolar disorder	10.51	7.94–14.10	<0.001*	7.84	4.26–13.53	<0.001*
Depression	1.65	1.55–1.76	<0.001*	1.99	1.68–2.35	<0.001*
Drug misuse	1.50	1.31–1.72	<0.001*	1.11	0.68–1.70	1.000
OCD	9.49	7.42–12.26	<0.001*	9.07	5.44–14.46	<0.001*
Psychosis	10.15	9.17–11.26	<0.001*	4.93	3.78–6.35	<0.001*
Schizophrenia	8.24	6.89–9.90	<0.001*	7.44	4.98–10.79	<0.001*
*Neurological conditions*						
Any studied neurological condition	1.48	1.38–1.58	*<0.001*	1.40	1.15–1.70	0.015*
Cluster headache	1.47	0.80–2.61	1.000	–	–	–
Epilepsy	9.21	7.99–10.64	<0.001*	7.15	5.19–9.66	<0.001*
Migraine headache	1.04	0.91–1.19	1.000	0.97	0.66–1.39	1.000
Tension headache	0.68	0.51–0.89	0.206	1.16	0.59–2.02	1.000

OCD, obsessive-compulsive disorder; bipolar, bipolar disorder; anxiety, generalised anxiety disorder plus phobias; ADHD, attention-deficit hyperactivity disorder.

Outcomes for associations with autism from binomial logistic regressions models incorporating age, sex, WIMD locality and all other conditions as co-variates. No cases of cluster headache in the extended cohort.

All significance figures adjusted for 30 tests. *Indicates significance at *p* < 0.05.

### Co-occurring neurological conditions

Of the general population control cohort, 3976 (15.32%) individuals' records included a neurological condition of interest, compared to 1617 (20.36%, χ^2^ = 111.6575, *p* ≤ 0.001) individuals' records in the core autism cohort, and 1774 (20.52%, χ^2^ = 125.9683, *p* ≤ 0.001) individuals' records with the extended autism phenotype. Epilepsy diagnosis was more likely to be recorded in hospital admission records (PEDW) than general practitioner (WLGP) healthcare records, whilst all headache diagnoses were the inverse (Table 2). Autism was associated with a higher prevalence of recorded epilepsy; core cohort participants were almost 10-fold more likely (OR 9.21, 95% CI 7.99–10.64) to have epilepsy on their healthcare records than general population control individuals after adjusting for age, sex and deprivation effects (Table 3).

Associations with headache subtypes were more complex. Migraine demonstrated no difference in frequency or odds ratio between the core, extended and control cohorts. Tension headache appeared at a lower prevalence in both the core (0.80%) and extended (0.86%) cohorts than in population controls (1.20%). Cluster headache appeared at a higher prevalence in both the core (0.20%) and extended (0.20%) cohorts than the general population (0.10%) controls, however the number of participants with this diagnosis was low and confidence intervals were wide (core cohort OR 1.47, 95% CI 0.80–2.61). No headache subtype risk model finding survived correction for multiple testing.

## Discussion

In this large population-based electronic-record case-control study, we used anonymised data from healthcare records to calculate prevalence estimates for psychiatric and selected neurological conditions within the adult autistic community. Our work replicates previous studies showing psychiatric problems are more prevalent in autistic adults than the general population (Hossain et al., 2020; Lai et al., 2019; Lugo-Marín et al., 2019). There are few comparable epidemiological studies of autistic adults drawn from the general population, with the majority of the literature examining clinical environment-derived cohorts (Hollocks et al., 2019). These clinical cohorts suggest around 70–90% of adults with ASD report a further lifetime psychiatric condition, though there exists a wide range of estimates from a variety of sample and study methodologies (Joshi et al., 2013; Pehlivanidis et al., 2020; Underwood et al., 2019). Lai et al.'s 2019 meta-analysis provides pooled estimates of co-occurring psychiatric conditions at 4–28% (Lai et al., 2019). Here we find co-occurring psychiatric diagnoses in the healthcare records of 47.63% of our core autism cohort, with autistic adults 2.83 times more likely to experience a co-occurring mental health condition. Two population birth cohorts, from Sweden and Minnesota, and an e-cohort derived from Californian health insurance data provide the closest study designs to our own and demonstrate comparable outcomes (Croen et al., 2015; Kirsch et al., 2020; Nimmo-Smith et al., 2020; Selten, Lundberg, Rai, & Magnusson, 2015). These demonstrated 54% of autistic adults had a psychiatric condition in Croen et al.'s study of healthcare insurance records, 20.1% of autistic adults had anxiety on their Swedish healthcare records in Nimmo-Smith et al.'s study, and US linked healthcare records in Kirsch et al.'s study finding cumulative incidence at 30 years of age in autistic adults of depression at 54.1%, anxiety at 50.0% and bipolar disorder at 7.3% (Croen et al., 2015; Kirsch et al., 2020; Nimmo-Smith et al., 2020).

ADHD is considered by some to be the most common co-occurring condition in autism, particularly in children, with prevalence estimates of 19–43% and a 2019 meta-analysis suggesting a pooled prevalence of 12.6% (Hofvander et al., 2009; Lugo-Marín et al., 2019; Simonoff et al., 2008). The low prevalence of lifetime ADHD diagnosis (7.00%) amongst our sample contrasts with previous literature (Pehlivanidis et al., 2020). This may be a product of our entirely adult population, or alternatively suggest diagnostic overshadowing or the presence of ADHD being a positive selection process for studies conducted in clinical populations. ADHD is predominantly diagnosed in childhood, and our study looking at records from adulthood are likely to miss some individuals, particularly where not transcribed into their electronic health records or receiving ongoing healthcare. A similar pattern is seen in OCD, with previously published figures from clinical samples of 11–37% not replicated by our population, where we observe a 3.02% adult prevalence (Joshi et al., 2013). Despite the low recorded prevalence within the core and extended autistic cohorts, ADHD and OCD both occur at significantly elevated prevalence when compared to the general population controls. Autistic adults within the core cohort were 10.74 times as likely to have an ADHD diagnosis on record and 9.49 as likely to have an OCD diagnosis on record than a general population control. Both ADHD and OCD are more frequently recorded in general practice (WLGP) healthcare data in this study, which may relate due to the low frequency of hospital admissions for either of these conditions, or under-recording and under-reporting on hospital healthcare records. The adult prevalence figure and odds ratio of 9.49 for OCD is comparable to the adjusted relative risk of 9.11 established in Nimmo-Smith et al.'s Swedish population birth cohort, further suggesting that previous studies using clinical samples derived from secondary care may be over-estimating general population prevalence (Nimmo-Smith et al., 2020).

The psychosocial effects and experiences of development as an autistic individual are known to be stressful, and underemployment, stigma and a lack of understanding are all potential contributors to development of anxiety or depression (Baldwin, Costley, & Warren, 2014; Howlin & Moss, 2012; Sperry & Mesibov, 2005). The odds ratios for anxiety (OR 1.90), depression (OR 1.65), alcohol (OR 1.25) and drug misuse (OR 1.50) were all elevated in autism although they were lower than for ADHD, bipolar disorder, OCD, psychosis and schizophrenia. Our findings of a higher prevalence of anxiety disorders (core cohort 22.40%) and OCD (core cohort 3.02%) are consistent with the aforementioned population studies of adults (Croen et al., 2015; Nimmo-Smith et al., 2020). Our results demonstrated a higher prevalence of depression (25.90%) than previous studies from the USA and Sweden (Croen et al., 2015; Kirsch et al., 2020). Anxiety, depression, alcohol misuse and drug misuse are all relatively prevalent in the general population, whilst ADHD, bipolar disorder, psychosis and schizophrenia are rarer and generally routinely managed with input from secondary care services. This pattern is observable in the source of diagnostic data, as depression and anxiety diagnoses occur in autistic individuals general practice records at much higher frequencies than in their hospital records. Given the disparity between the prevalence on hospital encounter record of anxiety (4.62%) and depression (6.62%) in autistic individuals and the published figures for the co-occurrence of these disorders it is highly likely these are an underestimate. We would propose that odds ratios for anxiety, depression, alcohol misuse and drug misuse are lower due to diagnostic overshadowing from autism, non-classical presentations in autistic individuals presenting diagnostic difficulties, or under-reporting or recording within the healthcare system (Kerns et al., 2015; Rosen, Mazefsky, Vasa, & Lerner, 2018; Stewart, Barnard, Pearson, Hasan, & O'Brien, 2006).

Cohort studies recruited from clinical environments suggest lifetime prevalence of psychosis in autism around 10–15%, though some smaller studies have suggested lower prevalence on clinical interview (Ghaziuddin & Zafar, 2008; Joshi et al., 2013; Stahlberg, Soderstrom, Rastam, & Gillberg, 2004; Underwood et al., 2019). Croen et al.'s 2015 analysis estimated the prevalence of schizophrenia at 8% in an autistic adult population (Croen et al., 2015), while Selten et al.'s 2015 study in Stockholm County, Sweden, found 0.6% of autistic youths (⩽17 years of age) had bipolar disorder and 0.6% non-affective psychotic disorder (Selten et al., 2015). Our estimate of recorded psychosis prevalence (18.30%) was higher than noted in previous literature, whilst the prevalence estimates for diagnosed bipolar disorder (2.50%) or schizophrenia (5.20%) were lower than recorded in adults. It has been noted that autistic individuals with psychosis can be misdiagnosed with a presentation of schizophrenia with negative symptoms (Skeppar et al., 2013). Given the significant genetic overlap and heterogeneity of presentation this may be unsurprising and points further towards the spectrum of presentations (Autism Spectrum Disorders Working Group of The Psychiatric Genomics Consortium, 2017).

Existing literature lacks a consensus around substance misuse in adults with autism. The prevailing hypothesis suggests that due to the core social and communicative aspects of autism, adults are less likely to engage in substance misuse (Rengit, McKowen, O'Brien, Howe, & McDougle, 2016). As a result the few population studies that have looked at substance misuse have estimated prevalence amongst adults with autism (0.90–2.19%) to be lower than the general population (3.92–11.90%) (Croen et al., 2015; Fortuna et al., 2015). However within the heterogeneity of autism presentations there appear to exist groups who are more at risk of substance or alcohol misuse, likely as self-medication for anxiety or distressing experiences (Lai et al., 2014). Qualitative studies and those examining clinically-referred samples would appear to include these populations, with accompanying increase in observations of alcohol and substance abuse or misuse (Hofvander et al., 2009; Lai et al., 2014; Underwood et al., 2019). Our results are not consistent with this overall picture. Amongst the Welsh population autistic adults demonstrated significantly elevated prevalence of and odds ratios for drug misuse and alcohol misuse compared to general population controls. The proportion of autistic adults with a diagnosed history of drug misuse was higher than those with a history of alcohol misuse, converse to the general population. The finding between these two so often co-occurring diagnoses is particularly interesting; whether this use represents periodic self-medication, a preference for specific substances instead of alcohol due to differing neurological effects and resultant experiences, or purely a difference of diagnostic practice is unclear.

Epilepsy has long been strongly associated with autism, as befits its neurodevelopmental nature (Lai et al., 2014; Pan, Bölte, Kaur, Jamil, & Jonsson, 2020; Rai et al., 2012). Epilepsy within autism appears to increase in frequency amongst those with intellectual disability or genetic syndromes. Prevalence estimates of 8–39% in clinical setting-derived samples are replicated in population studies, and in our own sample (core cohort 8.90%) (Croen et al., 2015; Howlin & Moss, 2012; Kohane et al., 2012; Lai et al., 2014). Though headache and migraine co-occur with anxiety and are seen in autism, few studies have examined the relationship, and those which do rarely differentiate subtypes (Canitano & Schröder, 2017). Croen et al. found no association with undifferentiated headache or migraine with ASD in their population (Croen et al., 2015). Here we find that undifferentiated headaches and tension headaches are recorded less frequently by adults with autism, consistent with a recent meta-analysis of neurological disorders in autism and contrary to an association with migraine identified in prior work (Pan et al., 2020; Underwood et al., 2019). No finding relating to headache survived correction for multiple testing.

This study analysed anonymised healthcare records from a nationally-representative, high-quality e-cohort of >3.6 million individuals. As with all e-cohort studies, we are unable to verify the diagnoses and therefore exposure and outcome misclassification cannot be excluded. Documentation prior to 2000 is limited due to the move from paper to electronic patient notes, and therefore individuals diagnosed prior to this point may have been missed by our methodology. In total, 21.67% of our sample had an autism diagnosis in both their WLGP (general practice) and PEDW (hospital) records, highlighting that there is a lack of reciprocity in records and our study may miss individuals with autism in the community who have not had contact with their GP or hospital services. This limitation is not confined to this study, with Hollocks et al.'s meta-analysis identifying that ‘there were no community studies that included adults whose ASD had not been recognised or who had not been in contact with clinical services’ (Hollocks et al., 2019). Indeed, previous work identifying autistic individuals in the SAIL Databank has suggested under-reporting or under-diagnosis (Underwood et al., 2021). Therefore, whilst useful for clinical services, this study is limited in generalisability to the whole autistic population, as individuals who have not presented to healthcare services and who may have lower rates of co-occurring conditions would not be included. Inaccurate documentation may lead to differential or non-differential misclassification, resulting in records not reflecting individuals' experiences or true outcome and an underestimation of effect sizes. For autistic individuals this may include inaccurate diagnoses due to historic lack of awareness, with alternative diagnoses being ascribed erroneously. A lack of standardised tools for diagnosis of co-occurring conditions in autism is well reported, with considerable variation in clinical practice (Hollocks et al., 2019; Nimmo-Smith et al., 2020). Different frequency of recording on general practice (WLGP) and hospital encounter (PEDW) of co-occurring disorders was also visible, suggesting under-recording in hospital records or potentially over-reporting in existing clinically-recruited estimates. Diagnostic overshadowing, the practice of one diagnosis taking primacy and leading to underdiagnosis of co-occurring conditions, is recognised to be a problem within autism (Hollocks et al., 2019; Kerns et al., 2015; Nimmo-Smith et al., 2020). This lack of recognition and diagnosis may represent an unmet need for these individuals, as they are not identified for support required, which may be compounded by social inequalities. We observed an increase in prevalence of autism in line with worsening deprivation, necessitating subsequent adjustment for deprivation score in our modelling. The effects of deprivation on prevalence of autism are debated, with links to greater parental education, race/ethnicity and social disadvantage (Bowden et al., 2020; Kelly et al., 2019; Maenner et al., 2021). Findings are clearer for the significant association between deprivation and other mental health conditions such as depression and substance misuse, as observed with risk of co-occurrence in the present study (Lee et al., 2020; Lorant et al., 2007; McLean et al., 2014; Remes et al., 2019).

As a study designed to estimate the prevalence of mental health disorders in autistic adults, we included records of only individuals aged ⩾18 years, and did not examine diagnostic codes exclusively observed in children and adolescents. Some neurodevelopmental and psychiatric conditions such as ADHD and certain subtypes of anxiety display greater incidence of diagnosis amongst children, using similar or the same diagnostic codes as in adulthood. The records of adults in this study were not back analysed into childhood, and therefore would not include entries in childhood including these diagnostic codes. Our estimates are likely to be an underestimate of full lifelong population prevalence for these conditions. These limitations may combine with those noted previously to produce errors in our estimates; however, the large sample size and replication of comparable previous results suggest these errors are not substantial. Where possible we have compensated through use of both primary and secondary care data. The limitations described are seen across e-cohort and record linkage methodologies which rely upon individuals' attendance, recognition and accurate recording.

In conclusion, in our nationally representative sample we replicate previous work demonstrating that psychiatric conditions co-occur at dramatically elevated prevalences amongst autistic adults than the general population, whilst adding to understanding about under-reporting and diagnostic overshadowing in this community. We confirm findings that the prevalence of epilepsy is increased in autistic adults. We find that in contrast to previous clinically-recruited studies, at a population level autism is associated with an increased prevalence of alcohol and drug misuse. Significant mental health conditions routinely managed in secondary healthcare services occur amongst autistic adults at 8.24–10.74 times the general population. We demonstrate that other mental health conditions often managed solely through primary care such as depression and anxiety occur with a high frequency but at lower odds ratios, which may relate to greater general population prevalence or under-reporting, recording or diagnostic overshadowing in hospital records. Co-occurring psychiatric conditions are common in autism, and with increasing numbers of adults being diagnosed with autism, services should be aware of the variety of presentations. Standardised and validated measures for use in the autistic population are needed to enable accurate diagnosis and further understand that shared underpinnings of psychiatric conditions and autism.

## Supplementary material

For supplementary material accompanying this paper visit https://doi.org/10.1017/S0033291722002884. click here to view supplementary material
