# Supplementary material for: Neurological and psychiatric disorders among autistic adults: a population healthcare record study
Source: Psychol Med. 2022 Oct 3;53(12):5663–73. doi: 10.1017/S0033291722002884 (PMC10482712; doi:10.1017/S0033291722002884)
Supplement: Supplementary file 1 [file S0033291722002884sup001.docx]

**Supplement One**

Contents

[Supplementary Tables 2](#_Toc109994163)

[Supplementary Table 1: Observed Frequency of Examined Mental Health Conditions in Extended Autism Phenotype Cohort with Statistical Analysis of Difference to Core Autism Phenotype 2](#_Toc109994164)

[Supplementary Table 2: Observed Frequency of Examined Mental Health Conditions in Combined Healthcare Records for Core Autism and General Population Control Cohorts, Stratified by Age 3](#_Toc109994165)

[Coding utilised for identification of extended autism spectrum phenotype in SAIL 4](#_Toc109994166)

[International Classification of Diseases – 10^th^ revision: 4](#_Toc109994167)

[Read Codes v2: 4](#_Toc109994168)

[International Classification of Diseases 10^th^ revision and Read Code version 2 coding list for co-occurring diagnoses: 5](#_Toc109994169)

# Supplementary Tables

## Supplementary Table 1: Observed Frequency of Examined Mental Health Conditions in Extended Autism Phenotype Cohort with Statistical Analysis of Difference to Core Autism Phenotype

| **Studied Condition** | **Observed Count of Diagnostic Codes** | **Percentage Proportion of Extended Cohort** | **Χ^2^ test statistic vs Core Cohort** | ***p* value** |
| --- | --- | --- | --- | --- |
| ADHD | 34 | 4.83% | 4.409 | *0.036* |
| Alcohol Misuse | 27 | 3.84% | 0.001 | 0.971 |
| Anxiety | 194 | 27.56% | 9.285 | *0.002* |
| Bipolar Disorder | 15 | 2.13% | 0.237 | 0.626 |
| Depression | 221 | 31.39% | 9.83 | *0.002* |
| Drug Misuse | 20 | 2.84% | 2.368 | 0.124 |
| OCD | 21 | 2.98% | <0.001 | 1 |
| Psychosis | 77 | 10.94% | 23.521 | *<0.001* |
| Schizophrenia | 34 | 4.83% | 0.095 | 0.758 |
| Epilepsy | 54 | 7.67% | 1.049 | 0.306 |
| Cluster Headache | 0 | 0.00% | - | - |
| Migraine Headache | 33 | 4.69% | 0.986 | 0.321 |
| Tension Headache | 11 | 1.56% | 3.65 | 0.056 |
| Headache (Unspecified) | 67 | 9.52% | 1.678 | 0.195 |

*Raw observed count frequency and calculated percentage prevalence of examined conditions. OCD = Obsessive Compulsive Disorder; Anxiety = Generalised Anxiety Disorder plus Phobias; ADHD = Attention Deficit Hyperactivity Disorder. Extended cohort denominator = 704. α = 0.05.*

## Supplementary Table 2: Observed Frequency of Examined Mental Health Conditions in Combined Healthcare Records for Core Autism and General Population Control Cohorts, Stratified by Age

|  | **Core Autism Phenotype Cohort** | | | | | **General Population Comparator Cohort** | | | | |
| --- | --- | --- | --- | --- | --- | --- | --- | --- | --- | --- |
|  | **18-25** | **26-35** | **36-50** | **51-65** | **65+** | **18-25** | **26-35** | **36-50** | **51-65** | **65+** |
| Any Studied Mental Health Condition | 1272 (32.02%) | 1199 (60.56%) | 777 (69.31%) | 412 (63.98%) | 123 (54.67%) | 1951 (16.08%) | 2290 (32.70%) | 1450 (37.84%) | 710 (32.03%) | 206 (27.21%) |
| ADHD | 314 | 178 | 48 | 14 | <10 | 129 | 45 | <10 | <10 | 0 |
| Alcohol Misuse | 45 | 113 | 103 | 42 | 10 | 145 | 306 | 234 | 117 | 31 |
| Anxiety | 503 | 614 | 419 | 200 | 47 | 948 | 1262 | 814 | 395 | 105 |
| Bipolar Disorder | 44 | 55 | 50 | 32 | 18 | 13 | 27 | 14 | <10 | <10 |
| Depression | 557 | 713 | 489 | 232 | 65 | 1254 | 1668 | 1083 | 508 | 124 |
| Drug Misuse | 84 | 134 | 84 | 21 | <10 | 159 | 292 | 212 | 47 | <10 |
| OCD | 52 | 81 | 63 | 34 | 10 | 19 | 37 | 24 | <10 | <10 |
| Psychosis | 338 | 444 | 379 | 215 | 77 | 108 | 192 | 183 | 97 | 20 |
| Schizophrenia | 59 | 118 | 130 | 71 | 33 | 37 | 48 | 56 | 28 | <10 |

*Raw observed count frequency of examined mental health conditions. Calculated percentage proportion for any studied mental health variable only due to low variable counts for specific diagnoses and SAIL reporting restrictions. OCD = Obsessive Compulsive Disorder; Anxiety = Generalised Anxiety Disorder plus Phobias; ADHD = Attention Deficit Hyperactivity Disorder. Core autism cohort denominator 7,943. Control cohort denominator 25,941.*

# Coding utilised for identification of extended autism spectrum phenotype in SAIL

## International Classification of Diseases – 10^th^ revision:

- F84 – Pervasive developmental disorders, containing:
  - F84.0 – Childhood autism
  - F84.1 – Atypical autism
  - F84.2 – Rett’s syndrome
  - F84.3 – Other childhood disintegrative disorder
  - F84.4 – Overactive disorder associated with mental retardation and stereotyped movements
  - F84.5 – Asperger’s syndrome
  - F84.8 – Other pervasive developmental disorders
  - F84.9 – Pervasive developmental disorders, unspecified

## Read Codes v2:

The following Read Codes v2 were selected to mirror the ICD-10 diagnostic coding:

- E140. – Autism/ infantile autism
- E1400 – Active infantile autism
- E1401 – Residual infantile autism
- E140z – Infantile autism NOS
- E141. – Disintegrative psychosis
- 1J9.. – Suspected autism
- E141. – Childhood disintegrative disorder
- Eu84. – Pervasive developmental disorder
- Eu840 – Childhood autism/ infantile autism
- Eu841 – Atypical autism
- Eu845 – Asperger syndrome
- Eu842 – Rett syndrome
- Eu843 – Other childhood disintegrative disorder
- Eu844 – Overactive disorder associated with mental retardation and stereotyped movements
- Eu845 – Asperger syndrome
- Eu846 – Pathological demand avoidance
- Eu84y – Other pervasive developmental disorder
- Eu84z – Pervasive developmental disorder, unspecified

# International Classification of Diseases 10^th^ revision and Read Code version 2 coding list for co-occurring diagnoses:

Please note, ‘% ‘ is a wildcard character to refer to the parent code and all its children in the hierarchy:

- ADHD: ICD-10 and Read codes from a list created for Langley e*t al.* as part of the All Wales Neurodevelopmental Disorder e-cohort (unpublished work) (*All Wales Neurodevelopmental Disorder E-Cohort - MRC Centre for Neuropsychiatric Genetics and Genomics - Cardiff University*, n.d.).
- ALCOHOL MISUSE: ICD-10 and Read code lists from DelPozo-Banos *et al*. (2018) (DelPozo-Banos *et al.*, 2018)
- ANXIETY
  - ICD-10 codes: F40% (Phobic anxiety disorders) and F41% (Other anxiety disorders)
  - Read codes from a revised list from Cornish *et al*. (2016)(Cornish *et al.*, 2016) (only diagnosis and symptoms) with addition of phobia codes (Eu40%, E202%)
- BIPOLAR DISORDER, PSYCHOSIS and SCHIZOPHRENIA 🡪 ICD-10 and Read code lists were created using as reference John *et al*. (2018) (John *et al.*, 2018); definition for ‘psychosis’ included codes for ‘bipolar disorder’, ‘schizophrenia’ and ‘other psychotic symptoms’.
- DEPRESSION
  - ICD-10 codes: F32% (Major depressive disorder single episode) and F33% (Major depressive disorder recurrent), excluding F323 and F333 because they refer to presence of psychotic symptoms; F341 (dysthymic disorder) and F412 (Mixed anxiety and depressive disorder).
  - Read codes from a revised list from Cornish *et al.* (2016) (Cornish *et al.*, 2016) (diagnosis and symptoms), plus extra codes 1BP0., Eu325, Eu326, Eu327, Eu32B
- DRUGS MISUSE: ICD-10 and Read code lists from DelPozo-Banos *et al.* (2018)(DelPozo-Banos *et al*., 2018)
- EPILEPSY
  - ICD-10 list was created referring to Jette *et al.* (2010) (Jette *et al.*, 2010) and contains chapters ‘G40.’ (Epilepsy) And ‘G41.’ (Status Epilepticus).
  - Read codes referred to Gorton *et al.* (2018) (Gorton *et al.*, 2018). Code lists were produced by searching for epilepsy-related terms in the CPRD medical dictionary. This list was refined following discussion with a neurologist and in comparison with studies previously conducted in the CPRD and the SAIL Databank. The epilepsy index date was defined as the latest date at which a person had received both an epilepsy diagnostic code plus an associated AED prescription. The AED prescription could be issued in the month before or up to 6 months after the date of the diagnostic code.
- HEADACHE (subgroups: CLUSTER HEADACHE, MIGRAINE, TYPE-TENSION HEADACHE, VASCULAR HEADACHE):
  - ICD-10 codes were based on Berg and further validated by a clinician (Berg, 2004).
  - Read codes were pulled out from the Read Browser v2 using as key words ‘headache’, ‘migraine’ and ‘cephalgia’. Following the indications of a clinician, codes denoting all iatrogenic type of headaches, psychogenic headaches and trigeminal headaches were excluded.
- OBSESSIVE-COMPULSIVE DISORDER: ICD-10 and Read code lists from DelPozo-Banos *et al* (DelPozo-Banos *et al.*, 2018)

# References:

*All Wales Neurodevelopmental Disorder e-cohort - MRC Centre for Neuropsychiatric Genetics and Genomics - Cardiff University*. (n.d.). Retrieved November 10, 2020, from https://www.cardiff.ac.uk/mrc-centre-neuropsychiatric-genetics-genomics/research/current-projects/all-wales-neurodevelopmental-disorder-e-cohort

Berg, J. (2004). Economic evidence in migraine and other headaches: A review. In *European Journal of Health Economics* (Vol. 5, Issue SUPPL. 1). https://doi.org/10.1007/s10198-005-0288-z

Cornish, R. P., John, A., Boyd, A., Tilling, K., & Macleod, J. (2016). Defining adolescent common mental disorders using electronic primary care data: a comparison with outcomes measured using the CIS-R. *BMJ Open*, *6*, 13167. https://doi.org/10.1136/bmjopen-2016

DelPozo-Banos, M., John, A., Petkov, N., Berridge, D. M., Southern, K., LLoyd, K., Jones, C., Spencer, S., & Travieso, C. M. (2018). Using Neural Networks with Routine Health Records to Identify Suicide Risk: Feasibility Study. *JMIR Mental Health*, *5*(2), e10144. https://doi.org/10.2196/10144

Gorton, H. C., Webb, R. T., Carr, M. J., DelPozo-Banos, M., John, A., & Ashcroft, D. M. (2018). Risk of unnatural mortality in people with epilepsy. *JAMA Neurology*, *75*(8), 929–938. https://doi.org/10.1001/jamaneurol.2018.0333

Jetté, N., Reid, A. Y., Quan, H., Hill, M. D., & Wiebe, S. (2010). How accurate is ICD coding for epilepsy? *Epilepsia*, *51*(1), 62–69. https://doi.org/10.1111/j.1528-1167.2009.02201.x

John, A., McGregor, J., Jones, I., Lee, S. C., Walters, J. T. R. R., Owen, M. J., O’donovan, M., DelPozo-Banos, M., Berridge, D., & Lloyd, K. (2018). Premature mortality among people with severe mental illness - New evidence from linked primary care data. *Schizophrenia Research*, *199*, 154–162. https://doi.org/10.1016/J.SCHRES.2018.04.009
